# Supplementary material for: GhBOP1 as a Key Factor of Ribosomal Biogenesis: Development of Wrinkled Leaves in Upland Cotton
Source: Int J Mol Sci. 2022 Sep 1;23(17):9942. doi: 10.3390/ijms23179942 (PMC9456263; doi:10.3390/ijms23179942)
Supplement: Supplementary file 1 [file ijms-23-09942-s001.zip › ijms-1873054-supplementary.pdf]

Supplementary:

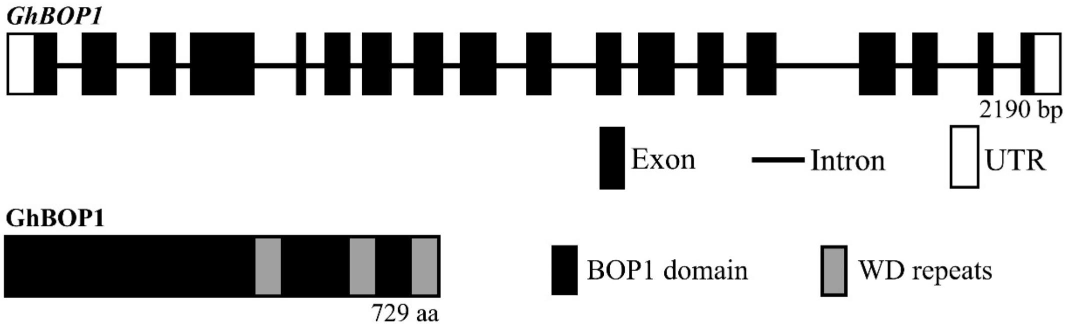

Figure S1. Genomic structure of GhBOP1, the exons and introns, and the predicted domains in GhBOP1.

Table S1. qPCR Primer Design

Gene Name	Gene ID	Fprimer	Rprimer
GhBOP1	Gh_A12G0013	TGCTGTACGAAGAAGATCGCCC	ACTTGGGCTTGAGCGATTCAGG
GhPES	Gh_A07G2047	GGACCCACCTCCACATCTGTCA	ACTCATCATTTCCCGTGCCAGG
GhWDR12	Gh_D09G0089	ACAACAGCCATTGCCATCCCTT	TTCCTCTTTTCGAGGGGCAACG
GhE2Fa	Gh_D01G1236	GAAGCCGCCATTTGTTCATCCG	AGACAGGTGTCTGGAACGGACT
GhE2Fb	Gh_A07G1021	ACTGAACCTGGGGTTGACTGGA	GCTCTCACTCCCGTTGTTGTGT
GhRBR	Gh_D04G1936	AAAAGATTGGAGAAAATA	ATCTCCGTACCCTCAAGT
GhCDC6	Gh_A03G0429	CCGATCGCAGTTCGTCTCCTTT	AGGGCTGCTTGGACTTGAATCT
GhORC1B	Gh_A08G0123	ATGCCAACAGAAACTCCT	TTTTCGAGGCGTGCTTAG
GhCDT1A	Gh_D11G1349	CCTTTGCTCCAACTCCTTCCCC	GCAGTCATCAGCCTAGCAGGAG
GhCDT1B	Gh_A11G1193	AGCACAGCCACAACCATCGAAT	TGGAGGCAGATCTGTAGGCACA
GhCYCD1;1	Gh_D02G1687	ACGAGGTGTTATCCGGCGATTC	CCGGGGACGAAGTTTGTCTCAT
GhCYCD3;1	Gh_D13G0418	GCGATGTGAGCATCTCCTCCTC	GGGCCTGGGGTCTTGTTGATAC
GhTAR2	Gh_D09G1630	ATGGCATACATATTGAGTGTTT	AGAGGAAGAGAGACGTGTTA
GhGH3.3	Gh_D11G1989	CAACTCTCTCCTCCGCAGTTGG	TGTCTCGGTCCGTAGCTCCATT
GhOPR3	Gh_A05G3948	TGATCACCTCGATGCAACCGAC	CCGTGATAGGTCCGCTTCAGTG

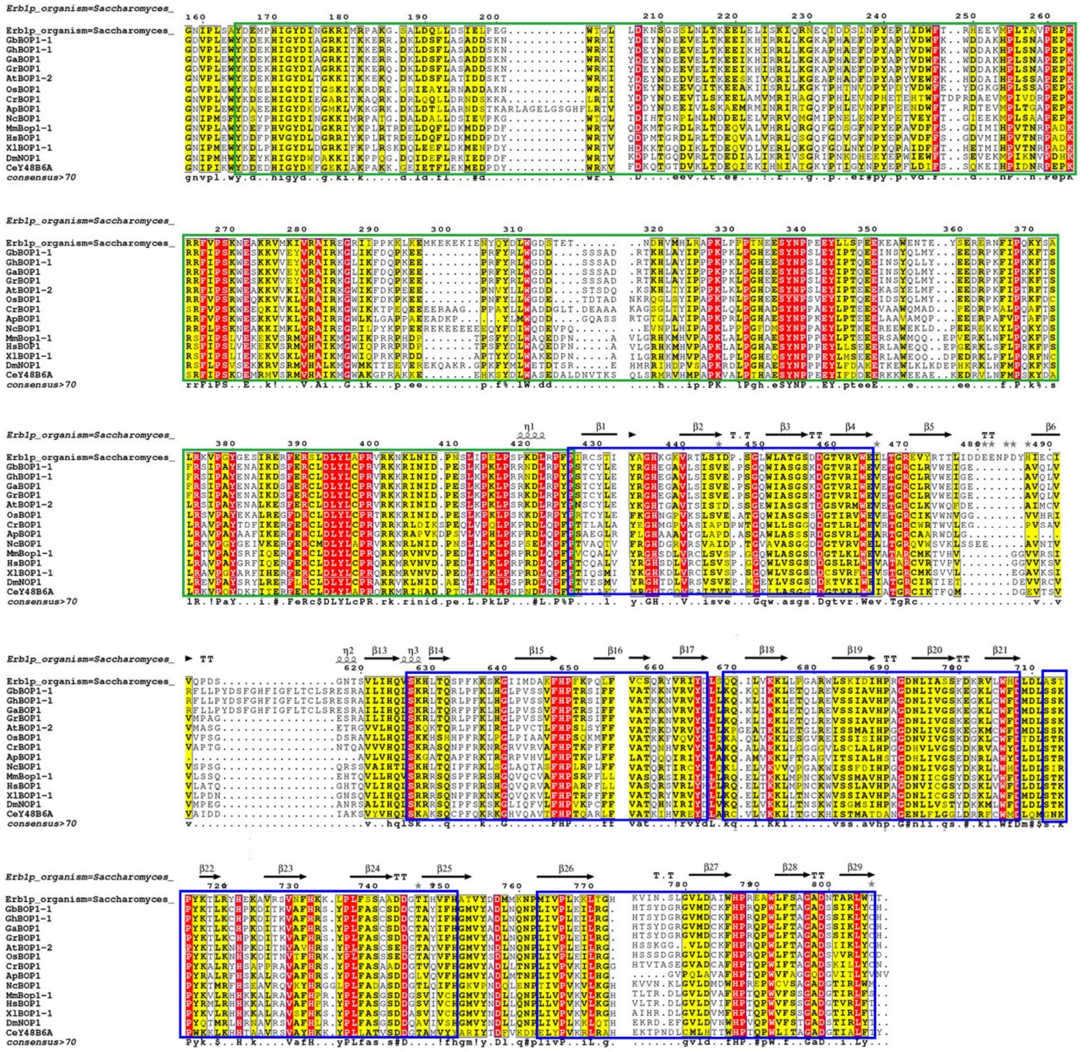

Figure S2. Sequence alignment analysis of BOP1 in different species. Highly conserved amino acid residues are represented by red boxes, the green boxes present the BOP1NT domain, and the blue boxes present the WD40 repeat sequence.
